# 

**Published:** 1879-03

**Authors:** 


### CONTENTS:

ARTICLE I.—Dental Neuralgia. By Dr. G. V. Black........................... 481

ARTICLE II.—Dr. J. Hall Moore's Address........................... 493

ARTICLE III.—Is Dentistry a Specialty of Medicine? By Dr. C. Stoddard Smith... 500

ARTICLE IV.—Virginia State Dental Association........................... 511

ARTICLE V.—Case of Oral Surgery. By H. M. Grant, D. D. S., M. D............. 519

### EDITORIAL, ETC.

The thirty-ninth Annual Commencement of the Baltimore College of Dental Surgery. 522

Annual Meeting of the Alumni Association of the Baltimore College of Dental Surgery........................... 525

### MONTHLY SUMMARY.

Good Effects of Ligation of the Thighs in Obstinate Epistaxis........................... 528

